# Increased Frequency of the Chemokine Receptor CCR2 on Circulating CD19+CD5+ Lymphocytes in Therapy‐Naïve CLL Patients With the Detectable EBV DNA and High‐Avidity Antibodies Against the EBV Capsid Antigen

**DOI:** 10.1155/cjid/3145486

**Published:** 2026-08-12

**Authors:** Laura Zvejniece, Svetlana Kozireva, Mariia Nazarenko, Olga Kornilova, Alla Rivkina, Sandra Lejniece, Ainars Leonciks, Modra Murovska, Elena Kashuba, Irina Kholodnyuk

**Affiliations:** ^1^ Institute of Microbiology and Virology, Riga Stradins University, Riga, Latvia, rsu.lv; ^2^ Clinic of Hematology, Riga East University Hospital, Riga, Latvia; ^3^ Department of Internal Diseases, Riga Stradins University, Riga, Latvia, rsu.lv; ^4^ Latvian Biomedical Research and Study Centre, Riga, Latvia, lu.lv; ^5^ Department of Microbiology, Tumor and Cell Biology, Karolinska Institutet, Stockholm, Sweden, ki.se; ^6^ Laboratory of Molecular Mechanisms of Cell Transformation, RE Kavetsky Institute of Experimental Pathology, Oncology and Radiobiology of National Academy of Sciences of Ukraine, Kyiv, Ukraine

**Keywords:** anti-EBV-capsid-antigen IgG avidity, BZLF1, CCR2, chronic lymphocytic leukemia (CLL), EBNA2, Epstein–Barr virus (EBV) load, LMP1

## Abstract

High EBV load in peripheral blood (PB) of untreated patients with chronic lymphocytic leukemia (CLL) has been associated with shorter overall survival. We demonstrated previously that EBV infection of B cells upregulates CCR1 and CCR2. For 54 therapy‐naïve CLL patients of the present study, we analyzed expression of CCR1, CCR2, and CD38 on circulating CD19+CD5+ lymphocytes, determined the EBV load (EBV DNA copy number) and the EBV transcripts (BZLF1, LMP1, LMP2A, and EBNA2) in PB mononuclear cells (PBMCs), and measured levels and avidity of IgG antibodies against the EBV capsid antigen (anti‐EBV‐CA‐IgG). The BZLF1 transcript was determined in one patient; both LMP1 and EBNA2 transcripts were detected in two patients only, and, in these patients, the frequency of CD19+CD5+ lymphocytes that presented CCR1 or CCR2 was elevated by 2.5‐, 4.3‐, and 2.9‐fold and by 2.1‐, 1.2‐, and 2.9‐fold, respectively, relative to the median values in the EBV‐positive group (> 5 EBV DNA copies in 10^5^ PBMCs; *n* = 21). The levels and avidity of anti‐EBV‐CA‐IgG were significantly higher in EBV‐positive patients compared with the EBV‐undetectable group (*p* = 0.007 and *p* = 0.018, respectively). Noteworthy, the high avidity of anti‐EBV‐CA‐IgG, with the relative avidity index (RAI) range 61.0–94.6, was associated in EBV‐positive patients with the increased 2.3‐fold frequency of CCR2 on CD19+CD5+ lymphocytes, compared to patients with the highest avidity (RAI > 95.0; *p* = 0.006). In patients with the highest anti‐EBV‐CA‐IgG avidity (RAI > 95.0; *n* = 13), indicative of the high rate of antigen presentation and a recent lytic viral reactivation, the frequency of the CCR2‐presenting CD19+CD5+ PB lymphocytes was 1.7‐fold lower than the median value of all high‐avidity patients (*n* = 31; *p* = 0.032). We hypothesize that CCR2 directs the migration of leukemic lymphocytes from circulation into ligand‐rich lymphoid organs, thereby underlying EBV’s contribution to CLL progression.

## 1. Introduction

Chronic lymphocytic leukemia (CLL), the most common type of adult leukemia/lymphoma in Europe and the United States, is a B‐cell neoplasm with a heterogeneous clinical course [1, 2]. Transformation of CLL to an aggressive lymphoma (Richter transformation) occurs in approximately 5% of patients, and the majority of these cases represent diffuse large B‐cell lymphoma (DLBCL) [3]. In a retrospective study of DLBCL Richter transformation, 58% of cases were clonally related to the initial CLL clone, and the presence of the Epstein–Barr virus (EBV)–encoded RNA (EBER) was documented in 5 of 85 cases (5.9%) [4]. Less than 1% of CLL patients developed biopsy‐proven classical Hodgkin lymphoma (cHL) in a retrospective study of 3887 CLL patients [5]. In patients with cHL of Richter transformation, 63−71% of cases were EBV‐positive [6, 7]. Impairment of immunity is common in CLL, leading to the increased incidence of infections and autoimmune complications and affecting the course of the disease [1, 8]. Several independent studies reported associations of high EBV DNA loads in PB cells of CLL patients at diagnosis with shorter overall survival (OS) and with a more aggressive clinical course of the disease [9–11].

The most relevant prognostic indicator in CLL is the mutation status of the immunoglobulin gene heavy chain variable region (*IGHV*) [2]. The unmutated *IGHV* (U‐IGHV) in leukemic cells had been associated with the more aggressive disease and shorter OS in CLL patients. The elevated presence of CD38 (cyclic ADP‐ribose hydrolase 1) on peripheral blood (PB) CLL cells (in more than 30% of cells) has been linked to the unfavorable prognosis and correlated with U‐IGHV in CLL [12–15]. In our earlier study of untreated CLL patients, we have shown that the presence of the inflammatory chemokine receptors CCR1 and/or CCR2 on circulating CD19+CD5+ cells positively correlated with the expression of the negative prognostic marker CD38 [16]. Inflammatory chemokines and their receptors regulate recruitment of immune cells and mediate migration and dissemination of malignant cells [17, 18].

In several studies, the detected EBV DNA in PB cells of CLL patients has been associated with the unfavorable prognostic factors, the elevated expression of CD38 on leukemic cells [9–11, 19], and the increased presence of the Zeta‐chain–associated protein kinase (ZAP70) [9, 11, 19]. EBV is a widespread human gamma herpesvirus with the main tropism for B cells. More than 90% of the general adult population are seropositive for antibodies against EBV antigens [20, 21]. In immunocompetent individuals, the virus establishes in memory B cells the latency (named Latency 0) downregulating all EBV proteins, which allows the virus to evade clearance by the host immune response and persist lifelong. Following differentiation of the memory B cells into plasma cells, the EBV persistence program (latency 0) switches to the lytic cycle (known as reactivation), producing infectious viral particles and enabling virus transmission. In immunocompetent hosts, the infectious virus is shortly cleared by the immune system [22, 23]. In the setting of immune suppression, EBV establishes in infected cells the latency III growth program that is characterized by the expression of all six EBV nuclear antigens (EBNAs) and both latent membrane proteins (LMP1 and LMP2). This type of latency III occurs in post‐transplant and HIV‐associated lymphomas, in EBV‐positive DLBCL, and in lymphoblastoid cell lines (LCLs) generated in vitro by infection of B cells with EBV. Latency II, where EBNA1, LMP1, and LMP2 are produced in the absence of other EBNAs, is usually found in EBV‐positive cHL, T/NK‐cell lymphomas, nasopharyngeal carcinoma, and DLBCL [22–24].

We have previously demonstrated that the in vitro EBV infection of B cells, isolated from PB of healthy donors, upregulated expression of the inflammatory chemokine receptors CCR1 and CCR2 but not CCR3 or CCR5 [25]. EBV is associated with various lymphomas of B‐, T‐, or NK‐cell origin, including Burkitt lymphoma (BL), DLBCL, and cHL. The role of EBV in CLL is considered mainly as a disease‐modifying factor, not as a cause of the disease. The virus’s preferential targets are B cells that express CD21, the major receptor for EBV [22, 23]. CLL cells express CD21 and can be infected by EBV in vitro. Nevertheless, in most cases, the ex vivo CLL cells infected by EBV underwent one‐two division cycles, but they did not proliferate [26].

First, we aimed to assess in the CLL patients prior to the treatment if the detectable EBV DNA in PB mononuclear cells (PBMCs) (> 5 copies per 10^5^ PBMCs) is associated with the high levels and the high avidity of the IgG antibodies against the EBV capsid antigen to presume a higher rate of antigen presentation (lytic viral reactivation). The aim of this study was to analyze in therapy‐naïve CLL patients a relationship between the elevated EBV loads or the presence of the EBV transcripts (BZLF1, LMP1, LMP2A, and EBNA2) in PBMCs and the cell‐surface expression of CCR1 and CCR2 on PB B‐ cells (CD19+CD5+ and CD19+CD5− lymphocytes). We also included in the statistical analyses the parameters of clinical examinations at the time of patient enrollment and the CLL disease negative prognostics, *IGHV* mutation status, *ZAP70* mRNA expression, and the CD38 positivity of PB B cells. We addressed the question whether EBV reactivation or the latent infection could stimulate migration of CLL lymphocytes from the PB into lymphoid organs, thereby promoting CLL disease progression.

## 2. Materials and Methods

### 2.1. Patients

Fifty‐four CLL patients, newly diagnosed during 2014–2020 in the Clinic of Chemotherapy and Hematology at Riga East University Hospital (REUH, Latvia), were included in the study: 24 females and 30 males with the age range 41–88 years (Table 1). The primary CLL diagnosis was made on the basis of an increase of blood B lymphocyte counts (> 5 × 10^9^/L) and co‐expression of the CD markers CD19/CD20, CD23, and CD5 on PB B cells [1, 2]. To characterize the clinical stages, the Rai classification [27] and its updated version [2] were applied. The study was approved by the Ethics Committee for Medical and Biomedical Research of Riga Stradins University (ethics approval No. 6‐A/14 from July 05, 2014, and ethics approval No. 6‐3/4/19 from April 25, 2019) and by the Central Medical Ethics Committee (ethics approval No. 01‐29.1/2734 from April 16, 2020). Written informed consent was obtained from all patients included in this study. Sample collection and the mutation status analysis of *IGHV* are described in Supporting Information (Supporting Methods).

**TABLE 1:** Demographic and clinical examination parameters in two groups of therapy‐naïve patients with chronic lymphocytic leukemia (CLL): the EBV DNA–positive and the EBV DNA–undetectable groups.

Demographic and clinical parameters	EBV‐positive (*n* = 21)	EBV‐undetectable (*n* = 33)	*p*‐value
Age (years) Median (IQR: Q1–Q3):	Range: 41–88 68.0 (64–74)	Range: 44–82 70.0 (61–75)	> 0.05

Gender: male, number (%): Female, number (%):	9 (42.9) 12 (57.1)	21 (63.6) 12 (36.4)	> 0.05

Low‐risk disease (Rai stage 0), number (%):	6 (28.6)	18 (54.6)	> 0.05

Intermediate‐risk disease (Rai stages I–II), number (%):	11 (52.4)	9 (27.3)	> 0.05

High‐risk disease (Rai III–IV), number (%):	4 (19.1)	6 (18.2)	> 0.05

TTFT (months) Median (IQR: Q1–Q3):	Range: 0.0–21.5 **0.0** (0.0–1.5)	Range: 0.0–60.0 **1.0** (0.5–7.0)	**0.015**

*IGHV* unmutated, number (%): *IGHV* mutated, number (%):	13 (61.9) 8 (38.1)	19 (57.6) 14 (42.4)	> 0.05

Leukocyte blood count (10^9^/L) Median (IQR: Q1–Q3):	Range: 10.2–148.0 22.0 (15.4–43.0)	(*n* = 32) Range: 8.1–142.7 20.5 (14.4–60.3)	> 0.05

Lymphocyte blood count (10^9^/L) Median (IQR: Q1–Q3):	(*n* = 20) Range: 6.1–81.0 20.0 (11.4–52.8)	(*n* = 29) Range: 5.4–103.0 15.4 (9.6–51.2)	> 0.05

Monocyte blood count (10^9^/L) Median (IQR: Q1–Q3):	(*n* = 18) Range: 0.2–12.9 1.0 (0.5–2.4)	(*n* = 29) Range: 0.3–16.1 0.7 (0.5–2.7)	> 0.05

Neutrophil blood count (10^9^/L) Median (IQR: Q1–Q3):	(*n* = 19) Range: 1.1–12.0 3.6 (3.2–4.7)	(*n* = 30) Range: 2.1–11.1 3.9 (3.0–4.7)	> 0.05

Platelet blood count (10^9^/L) Median (IQR: Q1–Q3):	Range: 57.0–274.0 175.0 (133.0–220.0)	(*n* = 31) Range: 101.0–313.0 167.0 (141.5–232.0)	> 0.05

Red blood cell count (10^12^/L) Median (IQR: Q1–Q3):	Range: 1.1–5.4 **4.1** (3.7–4.3)	(*n* = 32) Range: 3.0–5.2 **4.4** (4.1–4.7)	**0.019**

Hemoglobin (g/L) Median (IQR: Q1–Q3):	Range: 45.0–167.0 **125.0** (111.0–134.0)	(*n* = 32) Range: 89.0–162.0 **137.0** (123.8–145.3)	**0.047**

CD19+ cells in PBL (%) Median (IQR: Q1–Q3):	Range: 47.0–97.1 82.8 (76.0–90.0)	Range: 37.3–98.5 83.2 (72.5–92.4)	> 0.05

CD5+ in CD19+ PBL (%) Median (IQR: Q1–Q3):	Range: 26.0–94.1 72.8 (61.8–81.7)	Range: 22.2–98.1 58.3 (49.7–76.6)	> 0.05

CD23+ cells in PBL (%) Median (IQR: Q1–Q3):	Range: 24.8–83.1 72.2 (55.0–78.6)	Range: 10.8–87.4 58.4 (50.1–72.0)	> 0.05

*Note:* EBV‐positive, the EBV‐positive patient group, with the viral load > 5 EBV DNA copies per 10^5^ PBMCs that has been determined using the commercial quantitative real‐time PCR kit; EBV‐undetectable, the EBV DNA–undetectable patient group, with the EBV load < 5 EBV DNA copies per 10^5^ PBMCs (the threshold level stated in the kit). TTFT, the time to the first treatment in months, with the follow‐up period of 60 months. To compare differences between two groups of CLL patients, the Mann–Whitney test (including outliers) with the level of significance *p* < 0.05 was applied. The median values with interquartile ranges (IQR: 25th percentile and 75th percentile) and the absolute ranges are presented. Values of statistically significant differences between the groups are shown in bold.

Abbreviations: PBL, peripheral blood lymphocytes; PBMC, peripheral blood mononuclear cells.

### 2.2. EBV DNA Load Quantification

The EBV, a human herpesvirus 4, belongs to a family of Herpesviridae that comprises large, enveloped double‐stranded DNA viruses [22, 23]. Quantification of the EBV DNA copy number in PBMC DNA of newly diagnosed CLL patients was done using the commercial kit EBV Real‐TM Quant (Sacace Biotechnologies S.r.l., Como, Italy) with the detection limit of five EBV DNA copies per 10^5^ cells (or ∼8.3 EBV DNA copies per 1 μg of PBMC DNA). Real‐time PCR was performed according to the kit protocol, using the CFX96 Touch Real‐Time PCR Detection System (Bio‐Rad Laboratories Inc., Richmond, CA, USA).

### 2.3. Detection of the EBV Antibodies

The plasma levels of the IgG antibodies against the EBV capsid antigen (anti‐EBV‐CA) and the EBV nuclear antigen 1 (anti‐EBNA1) were determined using the commercial enzyme‐linked immunosorbent assay (ELISA) Euroimmun kits (EI 2791‐9601 G and EI 2793‐9601 G; Euroimmun Medizinische Labordiagnostika, Lubeck, Germany) that included the calibration samples and the positive and negative controls. Plasma samples were run in duplicate. The results were quantified, according to the kit’s instructions, and presented in relative units per mL (RU/mL). Absorbance was measured at 450 nm, using a Varioskan LUX multimode microplate reader (Thermo Fisher Scientific Inc.). The avidity of the anti‐EBV‐CA IgG antibodies was determined using the Euroimmun ELISA kit: Avidity Determination Anti‐EBV‐CA ELISA (IgG) (EI 2791‐9601‐1 G) and were expressed as the relative avidity index (RAI) that has been calculated out of the measured values with and without dissociating buffer incubation, according to the kit’s protocol. For avidity determination, plasma samples were run in 2 plates with positive, negative, high‐avidity, and low‐avidity controls. A commercial semiquantitative ELISA kit (Euroimmun EI 2791–9601 M) was used to measure in plasma samples the IgM antibodies against EBV‐CA. Prior to this ELISA test performance, all IgG subclasses and rheumatoid factors had been precipitated and removed from the samples, according to the kit’s protocol.

### 2.4. Detection of mRNA Expression of the EBV Genes BZLF1, LMP1, LMP2A, and EBNA2

The mRNA expression of the EBV immediate‐early lytic gene BZLF1 and the latent genes LMP1, LMP2A, and EBNA2 was assessed using the first‐strand cDNA prepared from the PBMC total RNA that was reverse‐transcribed with the random hexamer primers. Prior to the nested PCR, the first‐strand cDNAs were examined for the presence and quantity of the housekeeping gene *GUSB* (glucuronidase beta, RefSeqGene: NG_016197.1) cDNA, using real‐time PCR. All samples have been assayed by nested PCR in at least two independent experiments. We applied primers that had been published previously [28–30] and are shown in Table S1.

### 2.5. Flow Cytometry (FC) Analyses

Following the primary CLL diagnosis in the clinical FC laboratory at REUC, the PB samples were stained within 24 h with fluorochrome‐conjugated antihuman mouse monoclonal antibodies, as we described previously [16]. The presence of CD45‐Alexa Fluor 700 (clone HI30), CD19‐PE‐Cy5 or CD19‐Horizon BV786 (clone HIB19), CD5‐PE‐Cy7 (clone L17F12) or CD5‐BV510 (clone UCHT2), CD38‐Horizon V450 (clone HB7), CD14‐PE (clone MφP9), and CD191‐Alexa Fluor 647 (clone 53,504) or CD192‐Alexa Fluor 647 (clone 48,607) on the PBMC populations was assessed by seven‐color FC, using a BD FACSAriaIIIu analyzer and the Diva 8.2 software (Becton, Dickinson and Company, Franklin Lakes, NJ, USA). The fluorochrome‐conjugated antibodies were purchased from BD Biosciences. The gaiting strategies are described in Supporting Information (Supporting Methods).

### 2.6. Statistical Analyses

Statistical analyses between two groups were performed by the Mann–Whitney *U* test, a nonparametric unpaired 2‐tailed rank sum test, including outliers (the data were not normally distributed). The level of *p* < 0.05 was considered statistically significant. Statistical analyses were performed using SPSS 29.0.0.0 (IBM, Chicago, USA).

## 3. Results

### 3.1. EBV DNA Loads in PBMCs and Characteristics of Study Subjects

We quantified the number of the EBV DNA copies in PBMCs of 128 therapy‐naïve CLL patients that were randomly selected, using the commercial kit with the detection limit of 5 EBV DNA copies in 10^5^ cells (or ∼8.3 EBV DNA copies per 1 μg of PBMC DNA). In 47 patients (36.7%), EBV DNA has been detected. We included in the present study 54 CLL patients that have been additionally examined for the mRNA expression in PBMCs of the EBV genes: the early lytic gene BZLF1 and the latent genes, LMP1, LMP2A, and EBNA2. The 54 study patients were divided into two groups: 21 patients (38.9%) with > 5 EBV DNA copies per 10^5^ of PBMCs (EBV‐positive) and 33 patients (61.1%) without detectable EBV DNA in PBMCs (EBV‐undetectable).

For 54 study patients, we have collected the demographic (age, gender) and available clinical examination data at the time of enrollment: Rai clinical stage (updated according to the ERIC‐2018 recommendation [2]), complete blood count, hemoglobin level, and immunophenotype of PB lymphocytes (the percentages of CD19+, CD5+, and CD23+ lymphocytes). We have also determined the *IGHV* mutation status and measured the *ZAP70* mRNA expression levels in PBMCs. The summarized data (the median values with the interquartile ranges and the minimum–maximum ranges) are presented in Table 1. There were no statistically significant differences between the EBV‐positive and EBV‐undetectable groups regarding the demographic and clinical examination parameters, with the exception for the absolute count of red blood cells and the hemoglobin levels. Although the differences of the median values were not high, 4.1 versus 4.4 (10^12^/L) and 125.0 versus 137.0 (g/L), respectively, they were significant (*p* = 0.019 and *p* = 0.047). The time to the first treatment (TTFT) was shorter in the EBV‐positive patients compared to the EBV‐undetectable group (*p* = 0.015) and accounted for 0.0 months (with the range 0.0−21.5) and 1.0 months (with the range 0.0−60.0), correspondingly. The *ZAP70* mRNA levels and the *IGHV* mutation status did not differ significantly between these two groups of the study patients (Table 1).

Proportions (%) of the CCR1‐, CCR2‐, and CD38‐positive cells (CCR1+, CCR2+, and CD38+, respectively) within the CD19+CD5+ and CD19+CD5− PB lymphocytes were assessed for both groups of patients. We did not observe statistically significant differences between the EBV‐positive (*n* = 21) and EBV‐undetectable (*n* = 33) patients (Table 2).

**TABLE 2:** Expression of CCR1, CCR2, and CD38 on the peripheral blood B cells in two groups of therapy‐naïve patients with chronic lymphocytic leukemia (CLL): the EBV DNA–positive and the EBV DNA–undetectable groups.

Parameters	EBV‐positive (*n* = 21)	EBV‐undetectable (*n* = 33)	*p*‐value
Anti‐EBV‐CA IgG (RU/mL) Median (IQR: Q1–Q3):	(*n* = 19) Range: 96.0–307.4 **244.7** (198.8–281.6)	(*n* = 26) Range: 77.8–301.4 **188.5** (151.6–226.6)	**0.007**

Anti‐EBV‐CA IgG avidity (relative avidity index, RAI) Median (IQR: Q1–Q3):	(*n* = 18) Range: 65.1–131.3 **95,4** (88.6–98.9)	(*n* = 26) Range: 61.3–112.7 **89.0** (78.8–91.8)	**0.018**

Anti‐EBNA1 IgG (RU/mL) Median (IQR: Q1–Q3):	(*n* = 18) Range: 61.0–264.9 150.7 (101.6–181.9)	(*n* = 28) Range: 16.4–219.6 122.2 (66.3–166.1)	> 0.05

CD19+CD5+ PBL in PBMCs (%) Median (IQR: Q1–Q3):	70.5 (42.5–78.9)	50.4 (19.2–72.6)	> 0.05

CCR1‐expressing CD19+CD5+ PBL (%) Median (IQR: Q1–Q3):	Range: 1.5–45.6 10.6 (4.5–21.1)	Range: 0.5–56.7 13.3 (8.4–24.3)	> 0.05

CCR2‐expressing CD19+CD5+ PBL (%) Median (IQR: Q1–Q3):	Range: 1.5–37.4 12.7 (9.7–24.4)	(*n* = 32) Range: 0.4–69.5 12.7 (7.0–20.6)	> 0.05

CD38‐expressing CD19+CD5+ PBL (%) Median (IQR: Q1–Q3):	Range: 0.1–69.6 10.1 (1.3–25.5)	Range: 0.1–79.3 15.7 (5.3–34.0)	> 0.05

CD19+CD5– PBL in PBMCs (%) Median (IQR: Q1–Q3):	2.5 (1.2–8.6)	2.8 (1.3–7.4)	> 0.05

CCR1‐expressing CD19+CD5– PBL (%) Median (IQR: Q1–Q3):	(*n* = 18)^1^ Range: 0.5–51.9 15.9 (6.6–26.5)	(*n* = 28)^1^ Range: 0.8–65.7 16.5 (7.8–29.3)	> 0.05

CCR2‐expressing CD19+CD5– PBL (%) Median (IQR: Q1–Q3):	(*n* = 18)^1^ Range: 0.0–55.3 16.6 (8.9–26.0)	(*n* = 27)^1^ Range: 0.5–85.3 15.0 (6.0–37.2)	> 0.05

CD38‐expressing CD19+CD5– PBL (%) Median (IQR: Q1–Q3):	(*n* = 18)^1^ Range: 1.6–95.2 12.2 (4.8–24.3)	(*n* = 28)^1^ Range: 0.2–89.0 19.8 (7.2–41.8)	> 0.05

*Zap70* mRNA relative expression level in PBMCs: Median (IQR: Q1–Q3):	(*n* = 19) Range: 0.3–14.3 2.8 (2.0–3.9)	Range: 0.1–14.2 2.6 (1.3–4.2)	> 0.05

*Note:* EBV‐positive, the EBV‐positive patient group, with the viral load > 5 EBV DNA copies per 10^5^ PBMCs that has been determined using the commercial quantitative real‐time PCR kit; EBV‐undetectable, the EBV DNA–undetectable patient group, with the EBV load < 5 EBV DNA copies per 10^5^ PBMCs, (the threshold level stated in the kit). Anti‐EBV‐CA IgG, the level of the IgG antibodies against the EBV capsid antigen (EBV‐CA) in relative units per mL (RU/mL). Anti‐EBV‐CA IgG avidity, the avidity level of the IgG antibodies against EBV‐CA; RAI, relative avidity index. Anti‐EBNA1 IgG, the level of the IgG antibodies against the EBV nuclear antigen 1 (EBNA1) in relative units per mL (RU/mL).

Abbreviations: PBL, peripheral blood lymphocytes; PBMC, peripheral blood mononuclear cells.

^1^The presence of CCR1, CCR2, and CD38 on the CD19+CD5– lymphocytes was analyzed in the limited number of patients when the CD19+CD5– population accounted for > 1% of PBMCs. The median values with interquartile ranges (IQR: 25th percentile and 75th percentile) and the absolute ranges (minimum–maximum) are presented. To compare differences between two groups of CLL patients, the Mann–Whitney test (including outliers) with the level of significance *p* < 0.05 was applied. Values of statistically significant differences between the groups are shown in bold.

### 3.2. The Levels and Avidity of the Circulating EBV‐Specific Antibodies

We measured the levels (RU/mL) of circulating IgG antibodies against the EBV capsid antigen (EBV‐CA) and the latency antigen EBNA1, using the commercial quantitative ELISA kits. The verified results have been received for 45 out of the 54 patients in the study. All 45 patients were seropositive, as defined by high levels of the IgG antibodies against EBV‐CA (anti‐EBV‐CA‐IgG) and the presence of the IgG antibodies against EBNA1 (anti‐EBNA1‐IgG). To exclude a recent primary infection, we measured the presence of the IgM antibodies against EBV‐CA (anti‐EBV‐CA‐IgM), all 45 plasma samples were below the cutoff level of the control. The lack of anti‐EBV‐CA‐IgM and the high levels of anti‐EBV‐CA‐IgG and anti‐EBNA1‐IgG excluded a recent primary infection. Although the levels of anti‐EBNA1‐IgG, indicative of memory to latent infection, were higher in the EBV‐positive patients (150.7 vs. 122.2 RU/mL, the median values), the difference between groups was not statistically significant (Table 2).

The level of anti‐EBV‐CA‐IgG, which is expected to increase in response to an ongoing infection or a recent lytic viral reactivation, indeed, was higher in the EBV‐positive patients (244.7 RU/mL, the median values) than in the EBV‐undetectable group (188.5 RU/mL, the median values), and this difference was statistically significant (*p* = 0.007) (Table 2). Moreover, we observed that the RAI of anti‐EBV‐CA‐IgG in the EBV‐positive patients was significantly higher (*p* = 0.018) compared to the EBV‐undetectable patients—95.4 versus 89.9 (the median values), suggesting the higher rate of EBV‐CA presentation and a recent lytic viral reactivation (Table 2). The avidity data have been received for 44 patients out of the 54 in the study.

The RAI of anti‐EBV‐CA‐IgG was then used to stratify the EBV‐positive patients into the high‐avidity (*n* = 9, RAI 61.0–94.6) versus the highest‐avidity (*n* = 13, RAI > 95.0) (Tables 3 and S2). The highest‐avidity group included 9 EBV‐positive patients and 4 patients with the EBV load of < 5 copies/10^5^ PBMCs. No differences were observed between the EBV‐positive high‐avidity and the highest‐avidity groups in various clinical characteristics (Table S2). Nevertheless, the *IGHV* mutation status and the *ZAP70* mRNA relative expression levels (RELs) differed statistically significant, although modestly, between these groups: 88.9 versus 38.5% of patients with unmutated *IGHV* (*p* = 0.021) and 2.4 versus 2.5 REL (*p* = 0.021), respectively (Table 3).

**TABLE 3:** Expression of CCR1, CCR2, and CD38 on the peripheral blood B cells in two groups of therapy‐naïve CLL patients: the EBV DNA–positive patients with the high avidity of anti‐EBV‐CA IgG and the patients with the highest avidity of anti‐EBV‐CA IgG.

Parameters	Anti‐EBV‐CA IgG, RAI < 95.0 (range: 65.1–94.6) EBV‐positive (*n* = 9)	Anti‐EBV‐CA IgG, RAI > 95.0 (range: 96.2–131.3) EBV‐pos (*n* = 9) and EBV‐und (*n* = 4)	*p*‐value
Anti‐EBV‐CA IgG (RU/mL) Median (IQR: Q1–Q3):	Range: 96.0–307.4 267.3 (230.0–277.2)	Range: 132.8–306.7 240.4 (201.7–280.9)	> 0.05

Anti‐EBV‐CA IgG avidity (relative avidity index, RAI) Median (IQR: Q1–Q3):	Range: 65.1–94.6 **88.5** (87.1–93.3)	Range: 96.2–131.3 **103.2** (97.0–105.5)	**0.04** × **10** ^**−4**^

Anti‐EBNA1 IgG (RU/mL) Median (IQR: Q1–Q3):	(*n* = 8) Range: 73.9–218.0 160.5 (124.5–202.7)	Range: 61.0–264.9 150.5 (101.8–178.1)	> 0.05

*IGHV* unmutated, number (%): *IGHV* mutated, number (%):	**8 (88.9)** **1 (11.1)**	**5 (38.5)** **8 (61.5)**	**0.021**

CD19+CD5+ PBL in PBMCs (%) Median (IQR: Q1–Q3):	Range: 13.8–89.9 68.8 (27.0–70.8)	Range: 4.8–96.9 78.2 (61.5–87.3)	> 0.05

CCR1‐expressing CD19+CD5+ PBL (%) Median (IQR: Q1–Q3):	Range: 3.1–30.2 16.6 (4.5–22.0)	Range: 1.0–45.6 13.3 (6.0–16.0)	> 0.05

CCR2‐expressing CD19+CD5+ PBL (%) Median (IQR: Q1–Q3):	Range: 1.5–37.4 **24.6** (21.2–26.3)	Range: 1.7–22.7 **10.7** (7.1–12.7)	**0.006**

CD38‐expressing CD19+CD5+ PBL (%) Median (IQR: Q1–Q3):	Range: 1.3–69.6 15.6 (3.4–25.5)	Range: 0.1–63.0 2.5 (0.3–19.8)	> 0.05

CD19+CD5– PBL in PBMCs (%) Median (IQR: Q1–Q3):	Range: 0.8–59.1 1.6 (1.1–3.5)	Range: 0.2–43.2 2.7 (1.2–7.3)	> 0.05

CCR1‐expressing CD19+CD5– PBL (%) Median (IQR: Q1–Q3):	(*n* = 8)^1^ Range: 6.5–48.8 25.1 (15.9–33.4)	(*n* = 10)^1^ Range: 0.9–65.7 12.2 (6.8–30.7)	> 0.05

CCR2‐expressing CD19+CD5– PBL (%) Median (IQR: Q1–Q3):	(*n* = 8)^1^ Range: 15.0–55.3 25.8 (19.0–28.0)	(*n* = 10)^1^ Range: 2.2–47.9 15.4 (6.7–35.9)	> 0.05

CD38‐expressing CD19+CD5– PBL (%) Median (IQR: Q1–Q3):	(*n* = 8)^1^ Range: 3.2–30.0 23.8 (11.8–26.7)	(*n* = 10)^1^ Range: 0.9–95.2 9.1 (6.8–15.1)	> 0.05

*Zap70* mRNA relative expression level in PBMCs: Median (IQR: Q1–Q3):	(*n* = 8) Range: 0.6–3.9 **2.4** (2.0–3.1)	(*n* = 12) Range: 0.3–10.0 **2.5** (1.1–2.9)	**0.021**

*Note:* EBV‐pos, the EBV‐positive patient group, with the viral load > 5 EBV DNA copies per 10^5^ PBMCs that has been determined using the commercial quantitative real‐time PCR kit; EBV‐und, the EBV DNA–undetectable patient group, with the EBV load < 5 EBV DNA copies per 10^5^ PBMCs (the threshold level stated in the kit). Anti‐EBV‐CA IgG, the level of the IgG antibodies against the EBV capsid antigen (EBV‐CA) in relative units per mL (RU/mL). Anti‐EBV‐CA IgG avidity, the avidity level of the IgG antibodies against EBV‐CA; RAI, relative avidity index. Anti‐EBNA1 IgG, the level of the IgG antibodies against the EBV nuclear antigen 1 (EBNA1) in relative units per mL (RU/mL). *IGHV*, the immunoglobulin gene heavy chain variable region.

Abbreviations: PBL, peripheral blood lymphocytes; PBMC, peripheral blood mononuclear cells.

^1^The presence of CCR1, CCR2, and CD38 on the CD19+CD5– lymphocytes was analyzed in the limited number of patients when the CD19+CD5– population accounted > 1% of PBMCs. The median values with interquartile ranges (IQR: 25th percentile and 75th percentile) and the absolute ranges (minimum–maximum) are presented. To compare differences between two groups of CLL patients, the Mann–Whitney test (including outliers) with the level of significance *p* < 0.05 was applied. Values of statistically significant differences between the groups are shown in bold.

Noteworthy, the high avidity of anti‐EBV‐CA‐IgG (RAI 61.0–94.6) in the EBV‐positive patients (the high‐avidity patients; *n* = 9) was associated with the increased 2.3‐fold frequency of the circulating CCR2‐expressing CD19+CD5+ lymphocytes (24.6% vs. 10.7%, the median values), compared to 13 patients with the highest avidity (RAI > 95.0) of anti‐EBV‐CA‐IgG (*p* = 0.006) (Table 3). This association was also maintained (*p* = 0.011) between the EBV‐positive high‐avidity patients (*n* = 9) and the EBV‐positive highest‐avidity patients (*n* = 9), 24.6% vs. 11.5%, the median values (data not shown).

When we compared the highest‐avidity patients (*n* = 13; RAI > 95) with all high‐avidity patients (*n* = 31; RAI range: 61.3–94.6), we observed statistically significant differences, although modest, in the percentages of the circulating CD19+CD5+ cells that presented CCR2 or CD38. The percentages of the PB CD19+CD5+ cells, expressing CCR2 or CD38, were reduced by 1.7‐fold (10.7 vs. 17.6%, the median values; *p* = 0.032) and by 10.0‐fold (25.0% vs. 2.5%, the median values; *p* = 0.048), respectively, in the highest‐avidity group (*n* = 13) compared with the high‐avidity patients (*n* = 31) (Table 4). This observation is in line with our hypothesis: During the recent lytic EBV reactivation, the boosting of the anti‐EBV‐CA antibodies occurs, increasing the level and avidity of these antibodies; in parallel, the newly EBV‐infected CLL cells upregulated the cell‐surface expression of CCR2 and have migrated from the periphery into CCR2 ligand‐rich secondary lymphoid organs.

**TABLE 4:** Expression of CCR1, CCR2, and CD38 on the peripheral blood B cells in two groups of therapy‐naïve CLL patients, with the high avidity of anti‐EBV‐CA IgG (*n* = 31) and with the highest avidity of anti‐EBV‐CA IgG (*n* = 13).

Parameters	Anti‐EBV‐CA IgG, RAI < 95.0 (range: 61.3–94.6) EBV‐pos (*n* = 9) and EBV‐und (*n* = 22)	Anti‐EBV‐CA IgG, RAI > 95.0 (range: 96.2–131.3) EBV‐pos (*n* = 9) and EBV‐und (*n* = 4)	*p*‐value
Anti‐EBV‐CA IgG (RU/mL) Median (IQR: Q1–Q3):	Range: 77.8–307.4 198.6 (172.3–245.9)	Range: 132.8–306.7 240.4 (201.7–280.9)	> 0.05

Anti‐EBV‐CA IgG avidity (relative avidity index, RAI) Median (IQR: Q1–Q3):	Range: 61.3–94.6 **87.7** (79.4–90.6)	Range: 96.2–131.3 **103.2** (97.0–105.5)	**0.02** × **10** ^**−5**^

Anti‐EBNA1 IgG (RU/mL) Median (IQR: Q1–Q3):	(*n* = 30) Range: 16.4–218.0 129.9 (74.4–161.1)	Range: 61.0–264.9 150.5 (101.8–178.1)	> 0.05

*IGHV* unmutated, number (%): *IGHV* mutated, number (%):	**22 (71.0)** **9 (29.0)**	**5 (38.5)** **8 (61.5)**	**0.046**

CD19+CD5+ PBL in PBMCs (%) Median (IQR: Q1–Q3):	**57.5** (21.9–71.4)	**78.2** (61.5–87.3)	**0.041**

CCR1‐expressing CD19+CD5+ PBL (%) Median (IQR: Q1–Q3):	Range: 0.5–56.7 14.0 (5.2–24.5)	Range: 1.0–45.6 13.3 (6.0–16.0)	> 0.05

CCR2‐expressing CD19+CD5+ PBL (%) Median (IQR: Q1–Q3):	Range: 0.4–69.5 **17.6** (10.8–24.9)	Range: 1.7–22.7 **10.7** (7.1–12.7)	**0.032**

CD38‐expressing CD19+CD5+ PBL (%) Median (IQR: Q1–Q3):	Range: 0.1–69.6 **25.0** (7.2–37.0)	Range: 0.1–63.0 **2.5** (0.3–19.8)	**0.048**

CD19+CD5– PBL in PBMCs (%) Median (IQR: Q1–Q3):	1.9 (1.2–6.8)	2.7 (1.2–7.3)	> 0.05
CCR1‐expressing CD19+CD5– PBL (%) Median (IQR: Q1–Q3):	(*n* = 26)^1^ Range: 0.8–64.4 20.9 (7.2–30.1)	(*n* = 10)^1^ Range: 0.9–65.7 12.2 (6.8–30.7)	> 0.05

CCR2‐expressing CD19+CD5– PBL (%) Median (IQR: Q1–Q3):	(*n* = 26)^1^ Range: 0.5–85.3 25.6 (12.8–31.0)	(*n* = 10)^1^ Range: 2.2–47.9 15.4 (6.7–35.9)	> 0.05

CD38‐expressing CD19+CD5– PBL (%) Median (IQR: Q1–Q3):	(*n* = 26)^1^ Range: 0.2–71.2 21.8 (11.8–30.0)	(*n* = 10)^1^ Range: 0.9–95.2 9.1 (6.8–15.1)	> 0.05

*Zap70* mRNA relative expression level in PBMCs: Median (IQR: Q1–Q3):	(*n* = 30) Range: 0.1–14.2 2.6 (1.5–3.8)	(*n* = 12) Range: 0.3–10.0 2.5 (1.1–2.9)	> 0.05

*Note:* EBV‐pos, the EBV‐positive patient group, with the viral load > 5 EBV DNA copies per 10^5^ PBMCs that has been determined using the commercial quantitative real‐time PCR kit; EBV‐und, the EBV DNA–undetectable patient group, with the EBV load < 5 EBV DNA copies per 10^5^ PBMCs (the threshold level stated in the kit). Anti‐EBV‐CA IgG, the level of the IgG antibodies against the EBV capsid antigen (EBV‐CA) in relative units per mL (RU/mL). Anti‐EBV‐CA IgG avidity, the avidity level of the IgG antibodies against EBV‐CA; RAI, relative avidity index. Anti‐EBNA1 IgG, the level of the IgG antibodies against the EBV nuclear antigen 1 (EBNA1) in relative units per mL (RU/mL). *IGHV*, the immunoglobulin gene heavy chain variable region.

Abbreviations: PBL, peripheral blood lymphocytes; PBMC, peripheral blood mononuclear cells.

^1^The presence of CCR1, CCR2, and CD38 on the CD19+CD5– lymphocytes was analyzed in the limited number of patients when the CD19+CD5– population accounted > 1% of PBMCs. The median values with interquartile ranges (IQR: 25th percentile and 75th percentile) and the absolute ranges (minimum–maximum) are presented. To compare differences between two groups of CLL patients, the Mann–Whitney test (including outliers) with the level of significance *p* < 0.05 was applied. Values of statistically significant differences between the groups are shown in bold.

### 3.3. The High EBV Loads and mRNA Expression of the EBV Genes in PBMCs

**FIGURE 1:**
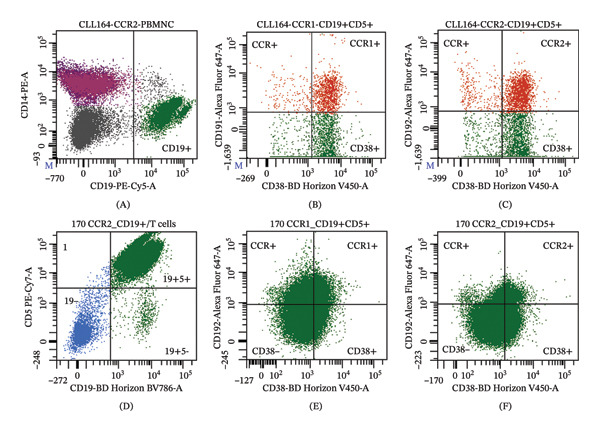
The cell‐surface expression of CCR1 and CCR2 on the peripheral blood (PB) CD19+CD5+ lymphocytes in two representative CLL patients with EBV transcripts in PBMCs. (A–C) The flow cytometry analysis of patient No. 164 with the high EBV load (258 DNA copies in 10^5^ PBMCs) and BZLF1 transcript detected in PBMCs. (D–F) The flow cytometry analysis of patient No. 170 with the mRNA co‐expression of LMP1 and EBNA2 in PBMCs and with the relative avidity index (RAI) > 95.0 of the plasma IgG antibodies against the EBV capsid antigen (EBV‐CA). Dot plots show the cells stained with antibodies targeting CD19 (anti‐CD19‐PE‐Cy‐5 or anti‐CD19‐Horizon BV786), CD14 (anti‐CD14‐PE), CD5 (anti‐CD5‐PE‐Cy7), CD38 (anti‐CD38‐Horizon V450), CCR1 (anti‐CD191‐Alexa Fluor 647), and CCR2 (anti‐CD192‐Alexa Fluor 647). A minimum of 20,000 gated cells were analyzed, and the threshold border was set at 1.0% of the stained cells. Cells were scored using a BD FACSAriaIIIu analyzer (Becton, Dickinson and Company, NJ, USA).

In 18 out of the 21 EBV‐positive patients, the number of the EBV DNA copies ranged from 5 to 46 per 10^5^ PBMCs. We detected the high EBV DNA load (> 200 copies/10^5^ PBMCs) in only three patients. Notably, in all three cases with the high EBV DNA load, two variants of *IGHV* were distinguished: unmutated and mutated *IGHV* (Table 5). The patient with 258 copies of EBV DNA (patient No. 164) was the only patient in whom the transcript of the EBV immediate‐early lytic gene BZLF1 was detected in PBMCs. In two patients with the high EBV load, No. 164 (258 DNA copies, the BZLF1 transcript‐positive) and No. 229 (with the highest EBV load of 523 DNA copies), the percentages of the PB CD19+CD5+ cells that expressed CCR1, CCR2, and CD38 were elevated by 2.5‐ and 2.7‐fold for CCR1+ (accounted for 26.8% and 28.4%, respectively), by 2.1‐ and 1.8‐fold for CCR2+ (accounted for 26.3% and 22.7%, respectively), and by 5.3‐ and 2.9‐fold for CD38+ (accounted for 53.0% and 29.6%, respectively), compared to the median values of the EBV‐positive patients (Tables 5 and S3; Figure 1B, C). In these two patients, the presentation of CCR1, CCR2, and CD38 on the PB CD19+CD5− lymphocytes was also enlarged (however, not so prominent as on the CD19+CD5+ cells) compared with the median values of the EBV‐positive group, by 1.7 and 1.5‐fold for CCR1+, by 1.6 and 1.3‐fold for CCR2+, and by 1.9 and 1.3‐fold for CD38+ (Table 5).

**TABLE 5:** Expression of CCR1, CCR2, and CD38 on the peripheral blood B‐cells of therapy‐naïve CLL patients with a high EBV DNA load in PBMCs (*n* = 3) and with the detected EBV transcripts BZLF1 (*n* = 1), and both LMP1 and EBNA2 (*n* = 2).

Parameters	EBV‐pos/EBV‐und	High EBV DNA load: copies/10^5^ PBMCs			LMP1 transcript‐positive patients	
	Median	BZLF1+			LMP2A–	LMP2A–
					EBNA2+	EBNA2+
EBV copies/10^5^ PBMCs:	16.0/< 5	264	258	523	6	6
Patient No		152	164	229	170	207
Anti‐EBV‐CA IgG (RU/mL):	**244.7/188.5**	282.4	307.4	306.7	132.8	230.0
Anti‐EBV‐CA IgG avidity (RAI):	**95.4/89.0**	87.1	87.1	99.1	108.2	93.3
Anti‐EBNA1 IgG (RU/mL):	150.7/122.2	73.9	169.3	69.4	150.5	151.7
*IGHV* status^#^ Variant 1/Variant 2 (% of mutations):	61.9/57.7 (% of U)	U (100/85.1)	U (100/87.1)	U (100/79.7)	M (82.4)	U (100)
CD19+CD5+ PBL in PBMCs (%):	70.5/50.4	13.8	30.1	65.8	78.2	13.8
CCR1‐expressing CD19+CD5+ PBL (%):	10.6/13.3	10.6	26.8	28.4	45.6	30.2
CCR2‐expressing CD19+CD5+ PBL (%):	12.7/12.7	13.0	26.3	22.7	15.1	37.4
CD38‐expressing CD19+CD5+ PBL (%):	10.1/15.7	10.1	53.0	29.6	33.7	69.6
CD19+CD5– PBL in PBMCs (%):	2.5/2.8	1.1	3.5	2.7	1.6	10.4
CCR1‐expressing CD19+CD5– PBL (%):	15.9/16.5	19.0	27.4	23.9	13.2	48.8
CCR2‐expressing CD19+CD5– PBL (%):	16.6/15.0	15.0	26.1	20.7	5.0	55.3
CD38‐expressing CD19+CD5– PBL (%):	12.2/19.8	24.8	22.8	15.6	10.7	29.8
Zap70 mRNA relative expression level in PBMCs:	2.8/2.6	4.0	0.6	10.0	2.8	2.87

*Note:* EBV‐pos, the EBV‐positive patient group, with the viral load > 5 EBV DNA copies per 10^5^ PBMCs that has been determined using the commercial quantitative real‐time PCR kit; EBV‐und, the EBV DNA–undetectable patient group, with the EBV load < 5 EBV DNA copies per 10^5^ PBMCs (the threshold level stated in the kit). EBV‐pos/EBV‐und, the median values for both CLL patient groups, the EBV DNA–positive and the EBV DNA–undetectable, are presented, and the values of statistically significant differences between the groups are shown in bold. Anti‐EBV‐CA IgG, the level of the IgG antibodies against the EBV capsid antigen (EBV‐CA) in relative units per mL (RU/mL). Anti‐EBV‐CA IgG avidity, the avidity level of the IgG antibodies against EBV‐CA; RAI, relative avidity index. Anti‐EBNA1 IgG, the level of the IgG antibodies against the EBV nuclear antigen 1 (EBNA1) in relative units per mL (RU/mL). *IGHV*, the immunoglobulin gene heavy chain variable region.

Abbreviations: PBL, peripheral blood lymphocytes; PBMC, peripheral blood mononuclear cells.

^#^For each CLL patient group (EBV‐pos and EBV‐und), the percentages of patients with unmutated *IGHV* are shown; U, unmutated *IGHV*; M, mutated *IGHV*. In three cases, two *IGHV* variants have been detected: percentages of total identity to the top germline V genes are presented for both variants in brackets. In PBMCs of patient No. 164 (with 258 EBV copies), the transcript of the EBV early lytic gene BZLF1 has been detected at the time of enrollment and flow cytometry analysis. Patient No. 229 (with 523 EBV copies), 18 months after the diagnosis with CLL, was diagnosed with the aggressive sebaceous carcinoma of the scalp with bilateral and abdominal metastases (T2N3M1 stage IVC); the patient underwent surgery and radiotherapy and died 24 months after the enrollment.

**FIGURE 2:**
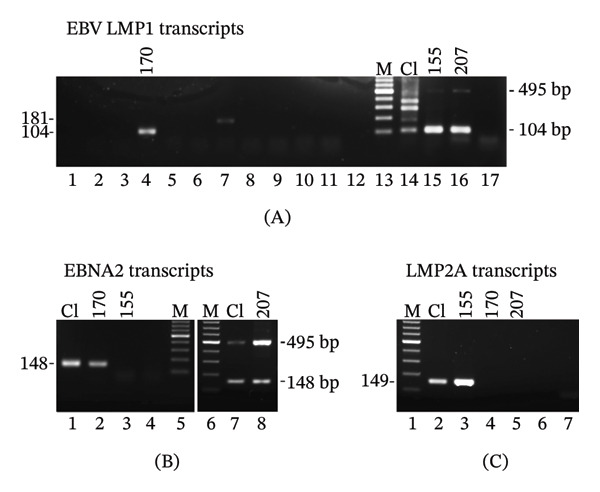
The mRNA expression of the EBV latent genes LMP1 and EBNA2 in the peripheral blood mononuclear cells (PBMCs) of therapy‐naïve CLL patients. The transcripts of LMP1, EBNA2, and LMP2A were determined using the generated first‐strand cDNA templates followed by nested‐PCR. (A) The EBV LMP1 transcripts (the PCR product of 104 bp) in PBMCs of CLL patients: lines 1–3 and 5–11, the LMP1 transcript‐negative CLL patients; line 4, the LMP1 transcript‐positive CLL patient No. 170; line 16, the LMP1 transcript‐positive patient No. 207; line 15, the LMP1 and LMP2A transcript‐positive patient with CD5‐positive B‐cell LPD; lines 12 and 17, a buffer; line 7, the LMP1 DNA PCR product of 181 bp is seen; Cl, the LMP1 transcript‐positive Burkitt lymphoma cell line BL16. Lines 15–17, duplex‐PCR: The first‐round cDNA templates were amplified with two pairs of primers, LMP1‐specific and the housekeeping gene *GAPDH*‐specific (the PCR product of 495 bp). (B) The EBV EBNA2 transcripts in PBMCs of CLL patients (the PCR product of 148 bp): line 2, the EBNA2 transcript‐positive CLL patient No. 170; line 3, the EBNA2 transcript‐negative patient No. 155 with CD5+ B‐cell LPD; line 4, a buffer; line 8, the EBNA2 transcript‐positive patient No. 207; Cl, the EBNA2 transcript‐positive lymphoblastoid cell line (LCL). Lines 7–8, duplex‐PCR: the first‐round cDNA templates were amplified with two pairs of primers, EBNA2‐specific and the housekeeping gene *GAPDH*‐specific (the PCR product of 495 bp). (C) The EBV LMP2A transcript has not been found in PBMCs of CLL patients but was detected in PBMCs of the LMP1 transcript‐positive patient No. 155 with CD5‐positive B‐cell LPD (the PCR product of 149 bp): line 3, the LMP2A transcript‐positive patient No. 155; line 4, the LMP2A transcript‐negative CLL patient No. 170; line 5, the LMP2A transcript‐negative CLL patient No. 207; line 7, a buffer; Cl, the LMP2A transcript‐positive LCL. M, GeneRuler 100 bp Plus DNA Ladder (Thermo Fisher Scientific Inc., Waltham, MA, USA).

The mRNA expression of the EBV latent genes, LMP1 and EBNA2, has been determined in PBMCs of two EBV‐positive patients only, with the low viral DNA load of 6 copies per 10^5^ PBMCs (Table 5). These two patients were both LMP1 and EBNA2 transcript‐positive (Figure 2). The LMP2A transcript has not been detected in the study patients. In two patients with the co‐expression of LMP1 and EBNA2 (No. 170 and No. 207), the percentages of the circulating CD19+CD5+ lymphocytes that presented CCR1, CCR2, and CD38 were considerably higher than the median values of the EBV‐positive patients: by 4.3‐ and 2.9‐fold for CCR1+ (accounted for 45.6% and 30.2%, respectively), by 1.2‐ and 2.9‐fold for CCR2+ (accounted for 15.1 and 37.4%, respectively), and by 3.3‐ and 6.9‐fold for CD38+ (accounted for 33.7 and 69.6, respectively) (Table 5; Figure 1E, F). The elevated proportions of the PB CD19+CD5− lymphocytes that expressed CCR1, CCR2, or CD38 were observed in only one LMP1/EBNA2 transcript‐positive patient No. 207. The second LMP1/EBNA2 transcript‐positive patient No. 170 was the highest‐avidity patient with an RAI of 108.2 (Tables 5 and S3).

## 4. Discussion

The high loads of EBV DNA in PB cells of untreated CLL patients have been previously associated with the shorter TTFT and OS and with the more aggressive clinical manifestation [9–11]. In our pilot study of 54 therapy‐naïve CLL patients that were randomly selected, we have also observed associations of the detectable EBV DNA in PBMCs (> 5 copies/10^5^ PBMCs) with the shorter TTFT and with the decreased concentrations of the red blood cells and hemoglobin. Moreover, we have demonstrated that the detectable EBV DNA (> 5 copies/10^5^ PBMCs) was significantly associated with the increased concentration of the IgG antibodies against EBV‐CA in plasma and with the increased avidity of these antibodies, thereby suggesting the higher rate of antigen presentation (lytic viral reactivation) in those patients.

The switch of the EBV latency programs to the lytic EBV reactivation can be induced in B cells by various stimuli and factors, including B cell receptor (BCR) activation [31], differentiation into plasma cells [32], hypoxia [33], transforming growth factor β (TGF‐β), temperature shifts, reactive oxygen species, and a variety of DNA damage agents (comprising also the chemotherapeutics) [31, 34]. Obviously, the activation of BCR by autoimmune anti‐BCR antibodies is a real scenario, considering the high rate of autoimmune manifestations in CLL. Following reactivation, the EBV immediate‐early lytic genes, BZLF1 (encodes the transcription factor Z) and BRLF1 (encodes the transcription factor R), are the first ones expressed. Expression of the transcription factors Z and R initiates the lytic cycle that results in production of infectious virion particles [24, 34]. An underlying T‐cell immunodeficiency in CLL patients may prolong the circulation period of the EBV virions produced in the lytic reactivation phase and, thus, prolong the period of the EBV‐CA presentation. Indeed, it was reported that the EBV load correlated positively with the expression of PD‐1 and its ligand PD‐L1 in CD4+ and CD8+ PB T cells in CLL patients [19], and expression of the immune‐checkpoint receptors, CTLA‐4 on CD3+ and CD86 on CD19+ PB lymphocytes, was significantly higher in the EBV DNA–positive than in EBV DNA–negative untreated CLL patients [35].

In our earlier study of untreated CLL patients, we have shown that the presence of CCR1 and/or CCR2 on circulating CD19+CD5+ cells positively correlated with the expression of the CLL negative prognostic marker CD38 [16]. Chemokine receptors and the corresponding chemokines ensure recruitment of immune cells and migration of cells via the mechanism of chemotaxis. Four chemokine receptors (CCRs), namely CCR1, CCR2, CCR3, and CCR5, belong to the family of inflammatory CCRs. CCR1 and CCR2 comprise significant protein sequence homology and share responses to the same chemokines [17, 18]. CCR2 responds to the chemokines CCL2, CCL7, CCL13, and CCL16, which are expressed at high levels in bone marrow (CCL2, CCL7), lymph nodes (CCL2, CCL13), spleen (CCL2), appendix (CCL7, CCL13), tonsil (CCL13), and liver (CCL2, CCL16) [36, 37].

Previously, we had demonstrated that the EBV infection of B cells, isolated from PB of healthy donors, upregulated expression of CCR1 and CCR2, but not CCR3 nor CCR5 [25]. The elevated mRNA and protein and the cell‐surface expression of both CCR1 and CCR2 were maintained in the established LCLs with EBV latency III and in the EBV‐positive BL cell lines with the type III growth phenotype [25, 38, 39]. Moreover, we have demonstrated the migration of LCL cells and BL B cells that co‐expressed LMP1 and EBNA2 toward the chemokine CCL2 (MCP1), the CCR2 dominant ligand [25, 38].

In the present study of 54 treatment‐naive CLL patients, we did not observe statistically significant differences between the EBV‐positive and EBV‐undetectable patients in the expression of CCR1 or CCR2 on CD19+CD5+ or on CD19+CD5− PB lymphocytes. However, in a patient with the high EBV load (523 DNA copies/10^5^ PBMCs) and in 3 patients with determined EBV transcripts, BZLF1 (in one patient) or both LMP1 and EBNA2 (in 2 patients), the percentages of the CCR1‐, CCR2‐, and CD38‐expressing CD19+CD5+ PB lymphocytes were notably higher than the median values of the EBV‐positive or the EBV‐undetectable patients.

Noteworthy, the high avidity of anti‐EBV‐CA‐IgG (RAI 61.0–94.6) in the EBV‐positive patients (*n* = 9) was associated (*p* = 0.006) with the increased 2.3‐fold frequency of the circulating CCR2‐expressing CD19+CD5+ lymphocytes, compared to patients with the highest avidity of anti‐EBV‐CA‐IgG (RAI > 95.0; 9 EBV‐positive and 4 EBV‐undetectable patients). This association was also maintained between the EBV‐positive high‐avidity patients (*n* = 9) and the EBV‐positive highest‐avidity patients (*n* = 9) (*p* = 0.011). We presume that the highest avidity (RAI > 95.0) indicates the recent lytic EBV reactivation, when, during production and circulation of the virions, the boosting of the antibodies against EBV‐CA was going on. At the same time, the EBV virions infected the neighboring CLL cells, inducing the cell‐surface expression of CCR1 and CCR2 and thus stimulating the migration of the newly infected CLL cells (CCR2‐expressing) from PB to the secondary lymphoid organs. Noteworthy, in the highest‐avidity patients (*n* = 13), the percentages of the CCR2‐expressing CD19+CD5+ cells in PB were 1.7‐fold lower (*p* = 0.032) than the median value of the high‐avidity patients (*n* = 31), including the EBV‐positive and the EBV‐undetectable patients.

The EBV preferential targets are B cells that express the major receptor for EBV—CD21 [22, 23]. CLL cells express CD21 and can be infected by EBV in vitro. Co‐activation of the EBV‐infected ex vivo CLL cells by cytokines and/or CD40L (anti‐CD40 antibodies) led to blast transformation and EBNA2 but not LMP1 expression (a state named latency IIb) [26]. Price and coauthors have demonstrated that latency IIb, when all of the EBNA proteins but not LMPs were expressed, was observed after EBV infection of ex vivo PB B cells during approximately 14 days before transition into latency III LCLs [40]. The single‐cell analysis, using immunohistochemistry, revealed heterogeneous expression of latency IIb in addition to latency III in the EBV‐associated lymphomas, post‐transplant lymphomas [41], and HIV‐associated lymphomas [42]. It has been suggested that in vivo, highly immunogenic LMP1 in the presence of functional cytotoxic T lymphocytes (CTLs) may favor selection for latency IIb over the latency III‐expressing cells [43]. The CLL cells with latency IIb in vitro underwent one‐to‐two division cycles; however, they did not proliferate [26]. Nevertheless, in rare cases, the EBV infection of ex vivo CLL cells produced proliferating cell lines that retained CD5 expression in culture and expressed both EBNA2 and LMP1 [44, 45]. LMP1 transcripts were found in PB, in 14% of cases in the cohort of 135 CLL patients that included untreated and post‐therapy patients, but only in 1% of the healthy control subjects (*n* = 98). In this cohort, the LMP1 positivity was significantly associated with the increased bone marrow involvement [46]. EBNA2 regulates the expression of the multiple cell transcription factors that promote B cell activation and enhance cell proliferation and transformation; LMP1 is a transformation protein that drives cell cycling and inhibits apoptosis in host cells [24]. LMP1 is found in exosomes secreted from the EBV‐infected cells and can be taken up by neighboring cells, altering their proliferation [47].

In the context of our study, we do not know which PBMCs contained the virus. EBV has been found in vivo in T and NK lymphocytes in the EBV‐positive T/NK‐cell lymphomas [48], in macrophages and dendritic/Langerhans cells in cHL, and in monocytes in HIV‐infected individuals [49]. Nevertheless, the high EBV DNA loads in PB cells of untreated CLL patients have been significantly associated with the shorter TTFT and OS [9–11]. In the study of 243 CLL patients at diagnosis, EBV DNA was detected in the whole blood in 10% of cases, and EBV DNA positivity was associated with therapy response, shorter TTFT, worse OS (HR 2.77), and a greater risk of Richter transformation [11]. A recent report demonstrated that CLL patients under therapy progressed into DLBCL (Richter transformation) driven by EBV reactivation [50]. However, the role of EBV infection and impaired T‐cell immunity in Richter transformation remains to be further investigated. High EBV DNA load in PBMCs of 220 prospective patients at CLL presentation (in 19% of the cases) led to a 3.14‐fold increase in the hazard ratio (HR) of death, and the multivariate analysis selected the high levels of EBV DNA load at presentation as an *IGHV* mutation status‐independent predictor of 5‐year OS in CLL patients. Notably, in this study, EBV DNA was detectable in the sorted CLL cells at the same levels as in PBMCs [10].

We can speculate that EBV may latently infect a number of CLL cells, and in CLL patients with deregulated immunity, the lytic EBV reactivation occurs repeatedly due to stimulation of BCR. Considering the results of the current pilot study and our previously reported data, we are proposing a possible mechanism by which EBV could contribute to CLL disease progression: Following the lytic virus reactivation, the EBV infection of PB CLL lymphocytes induces the cell‐surface expression of CCR2, which directs the migration of leukemic lymphocytes from the circulation into CCR2 ligand‐rich lymphoid organs. Further investigations will advance our understanding of the role EBV could play in CLL.

EBV‐directed cellular immunotherapies that include EBV‐specific cytotoxic T lymphocytes (EBV‐CTLs), engineered allogeneic T‐cell receptor T cells (TCR‐T), and chimeric antigen‐receptor T cells (CAR‐T) have been tested in early‐phase trials for EBV‐positive post‐transplant lymphoproliferative disorders (PTLDs) and EBV‐associated malignancies: nasopharyngeal carcinoma, HL, and NK/T‐cell lymphoma. Although the EBV‐directed cellular immunotherapies have demonstrated promising efficacy in clinical trials, the clinical implementation of these immunotherapies faces a number of limitations, including toxicities due to cytokine release syndrome, immune evasion mediated by EBV, and heterogeneity in the immune status of patients [51, 52]. A new strategy of therapy for EBV‐positive malignancies involves the induction of EBV lytic reactivation combined with antiviral drug (ganciclovir/valganciclovir) treatment. The lytic‐induction therapy plus antiviral drugs has been explored in preclinical and early‐phase studies in several EBV‐positive malignancies, including PTLD, HL, and DLBCL [51, 53, 54]. Currently, one registered clinical trial phase 2 study, “EBV Lytic Reactivation Therapy Combined With PD‐1 Antibody in Recurrent/Metastatic Nasopharyngeal Carcinoma” [55], started in 2025, is using a lytic‐induction approach (https://clinicaltrials.gov/study/NCT07138989). There is no available evidence yet that an EBV‐directed therapy improves survival in EBV‐positive CLL. Understanding of the EBV biology and underlying immunodeficiency in CLL may advance therapeutic strategies to enhance the efficacy and safety of the treatment.

Limitations of this report include the relatively small number of patients, although broadly investigated. Nevertheless, the statistical analysis has revealed statistically significant differences between the selected groups. In this study, we examined only EBV, though there is evidence that another herpes virus, cytomegalovirus (CMV), may play a role in immunosuppressed patients with CLL [56]. Further studies, using a larger number of patients and comparison with CMV, will be required to investigate the role of EBV/CMV reactivation in the pathogenesis of CLL.

## Author Contributions

Irina Kholodnyuk, Laura Zvejniece, and Svetlana Kozireva conceived and designed the study and analyzed the data. Irina Kholodnyuk, Laura Zvejniece, Svetlana Kozireva, and Mariia Nazarenko performed research. Alla Rivkina and Olga Kornilova collected samples and clinical data. Alla Rivkina, Sandra Lejniece, and Modra Murovska curated clinical data and provided advice in interpreting the results. Irina Kholodnyuk supervised the projects and wrote the manuscript. Ainars Leonciks and Elena Kashuba helped with analyzing data and revising the manuscript. All authors discussed the results and commented on the manuscript.

## Funding

This study was supported by the Latvian Council of Science (Grants Nos. 651/2014 and lzp‐2018/1‐0156) and by Riga Stradins University (Grant No. 6‐ZD‐22/14/2022).

## Disclosure

The funders had no role in the design of the study; in the collection, analyses, or interpretation of data; in the writing of the manuscript; or in the decision to publish the results. All authors approved the final version.

## Ethics Statement

This study was approved by the Institutional Ethics Committee for Medical and Biomedical Research (Riga Stradiņš University, Latvia) (ethics approval No. 6‐A/14 from 05 July 2014 and ethics approval No. 6‐3/4/19 from 25 April 2019) and by the Central Medical Ethics Committee (Latvia) (ethics approval No. 01‐29.1/2734 from April 16, 2020), authorizing publications of anonymized clinical and patient data. The study was conducted in accordance with the Declaration of Helsinki. Informed consent was obtained from all patients involved in the study.

## Consent

Please see the Ethics Statement.

## Conflicts of Interest

The authors declare no conflicts of interest.

## Supporting Information

Additional supporting information can be found online in the Supporting Information section.

## Supporting information


**Supporting Information** Supporting Methods: 1.1. Sample Collection and Processing. 1.2. Mutation Status Analysis of the Immunoglobulin Gene Heavy Chain Variable Region (IGHV). 1.3. Detection of the ZAP70 mRNA expression levels. 1.4. Flow Cytometry Analyses. Table S1. The EBV primers used for the RT‐PCR analysis. Table S2. Demographic and clinical examination parameters in two groups of therapy‐naïve CLL patients: in the EBV DNA–positive patients with the high avidity of anti‐EBV‐CA IgG and the patients with the highest avidity of anti‐EBV‐CA IgG. Table S3. Demographic and clinical examination parameters of therapy‐naïve CLL patients with high EBV DNA load in PBMCs (*n* = 3) and with the detected EBV transcripts, BZLF1 (*n* = 1) and LMP1 and EBNA2 (*n* = 2). References to the Supporting Information.

## Data Availability

The data that support the findings of this study are deposited into the institutional repository Dataverse (https://dataverse.rsu.lv/) and are available upon request to the corresponding author (doi: https://doi.org/10.48510/FK2/LNBXIU).
