# Supplementary material for: Increased Frequency of the Chemokine Receptor CCR2 on Circulating CD19+CD5+ Lymphocytes in Therapy‐Naïve CLL Patients With the Detectable EBV DNA and High‐Avidity Antibodies Against the EBV Capsid Antigen
Source: Can J Infect Dis Med Microbiol. 2026 Aug 12;2026:3145486. doi: 10.1155/cjid/3145486 (PMC13463309; doi:10.1155/cjid/3145486)
Supplement: Supplementary file 1 [file CJID-2026-3145486-s001.docx]

**Supplementary Material**

1. Supplementary Methods: 1.1. Sample Collection and Processing. 1.2. Mutation Status Analysis of the Immunoglobulin Gene Heavy Chain Variable Region (IGHV). 1.3. Detection of the ZAP70 mRNA expression levels. 1.4. Flow Cytometry Analyses.
2. Table S1. The EBV primers used for the RT-PCR analysis.
3. Table S2. Demographic and clinical examination parameters in two groups of therapy-naïve CLL patients, in the EBV DNA-positive with the high avidity of anti-EBV-CA IgG and in the patients with the highest avidity of anti-EBV-CA IgG.
4. Table S3. Demographic and clinical examination parameters of therapy-naïve CLL patients with high EBV DNA load in PBMCs (n=3) and with the detected EBV transcripts, BZLF1 (n=1), LMP1 and EBNA2 (n=2).
5. References to the Supplementary Material.

**1. Supplementary Methods**

**1.1. Sample Collection and Processing**

Blood was collected into sterile BD Vacutainer tubes containing EDTA (Thermo Fisher Scientific) the same time as assessment of the primary diagnosis in the clinical flow cytometry (FC) laboratory at REUC. Within 24 hours, PBMCs and plasma were isolated using Histopaque-1077 (Sigma-Aldrich, Germany) density gradient. Plasma supernatant was obtained by centrifugation, aliquoted, and stored at -80°C prior to use. PBMCs, after density gradient centrifugation, were washed with PBS supplemented with 2% fetal bovine serum (FBS), counted, and divided into two parts. One part of each sample was further processed with TRI Reagent (Sigma-Aldrich, Germany) for RNA extraction, according to the manufacturer’s protocol. The second parts of PBMCs (2–5 × 10^6) were stored at -80°C prior to the genomic DNA isolation with phenol-chloroform extraction and ethanol precipitation (<https://doi.org/10.1002/0471142905.hga03cs00>).

**1.2. Mutation Status Analysis of the Immunoglobulin Gene Heavy Chain Variable Region (IGHV)**

The *IGHV* mutation status was defined according to the International CLL IPI working group (iwCLL) and the European Research Initiative on CLL (ERIC) recommendations [Hallek et al., 2018], applying the ERIC published protocol [Agathangelidis et al., 2018]. The protocol includes the amplification of the *IGHV* DNA, using multiplex PCR with nine 5’ *IGHV* leader primers and four 3’ *IGHJ* joining region primers, to cover the complete variable region, and the direct sequencing of the isolated PCR-products. The 98-100% sequence total identity to the top germline V genes, according to the NCBI human Germline V gene Database and IGBLAST (<https://www.ncbi.nlm.nih.gov/igblast/>), was considered as unmutated *IGHV* (*U-IGHV*).

**1.3. Detection of the ZAP70 mRNA expression levels**

The *ZAP70* (RefSeqGene: NG_007727.1) mRNA expression levels were assessed in PBMC pools, applying the real-time PCR analysis and a protocol that was described previously [Kholodnyuk et al., 2020; Gladkikh et al., 2017]. The *ZAP70* values were normalized to the values of the B-cell housekeeping gene *GUSB* (glucuronidase beta, RefSeqGene: NG_016197.1). We used one patient cDNA sample, with the low-moderate level of *ZAP70* mRNA expression, as an inter-run calibrator (IRC).

**1.4. Flow Cytometry Analyses**

Following the primary CLL diagnosis in the clinical flow cytometry (FC) laboratory at REUC, the PB samples were stained within 24 hours with fluorochrome-conjugated anti-human mouse monoclonal antibodies as we described previously [Kholodnyuk et al., 2020]. The presence of CD45-Alexa Fluor 700 (clone HI30), CD19-PE-Cy5 or CD19-Horizon BV786 (clone HIB19), CD5-PE-Cy7 (clone L17F12) or CD5-BV510 (clone UCHT2), CD38-Horizon V450 (clone HB7), CD14-PE (clone MφP9), CD191-Alexa Fluor 647 (clone 53504) or CD192-Alexa Fluor 647 (clone 48607) on the PBMC populations was assessed by seven-color FC, using a BD FACSAriaIIIu analyzer and the Diva8.2 software (Becton, Dickinson and Company, Franklin Lakes, NJ, USA). Apoptotic cells were excluded from the analyses via the staining with AnnexinV-PerCP-Cy5.5. As the negative controls, nonspecific mouse IgG1 and IgG2b, matching the Ig-isotype of CD191 (CCR1) and CD192 (CCR2), were used. The fluorochrome-conjugated antibodies were purchased from BD Biosciences. Applying the gating on viable cells and SSC/FSC dot-plots, the CD45+ leukocytes were divided into granulocytes and the PBMC sets: the monocyte (CD14+) and lymphocyte populations. The CD19-positive lymphocytes were further separated into two sub-populations, the CD5-positive (CD19+CD5+) and CD5-negative (CD19+CD5–). The percentages of the CCR1-, CCR2-, and CD38-presenting lymphocytes have been counted in each sub-population.

**Table S1.** The EBV primers used for the RT-PCR analysis.

| EBV gene | Symbol | Sequences (5’ – 3’) | Position within the EBV RefSeq NC_007605.1. | Exon | cDNA size (bp) | Reference |
| --- | --- | --- | --- | --- | --- | --- |
| BamHI Z fragment leftward open reading frame 1  (outer primers) | BZLF1 | GGCAGCAGCCAC/CTCACGGT  TTCCACAGCCTGCACCAGTG | 90042–90053/90138–90145  90431-90412 | 3 / 2  1 | – | Bell et al, 2006; Tierney et al, 1994 |
| BamHI Z fragment leftward open reading frame 1  (inner primers) | BZLF1 | GGCAGCAGCCAC/CTCACGGT  ACGACGCACACGGAAACC | 90042-90053/90138-90145  90400-90383 | 3 / 2  1 | 151 | Bell et al, 2006 |
| Latent membrane protein 1 (outer primers) | LMP1 | GTCATAGTAGCTTAGCTGAAC  CTTCAGAAGAGACTTTCTCT | 167705‒167725  168804‒168785 | 1 / 3 | - | Tierney et al, 1994; Neves et al, 2015. |
| Latent membrane protein 1  (inner primers) | LMP1 | CTTCAGAAGAGACCTTCTCT  ACAATGCCTGTCCGTGCAAA | 168804‒168785  168623‒168642 | 1 / 2 | 104 | Tierney et al, 1994 |
| Epstein-Barr nuclear antigen 2  (outer primers) | EBNA2 | AGAGGAGGTGGTAAGCGGTTC  TGACGGGTTTCCAAGACTATCC | 14802‒14822  36296‒36275 | 16  1 | - | Tierney et al, 1994; Bell et al, 2006. |
| Epstein-Barr nuclear antigen 2  (inner primers) | EBNA2 | GCTTAGCCAG/TAACCCAGCACT  GCAAGATAGAATGTAGGCAT | 35702‒35711/36098‒36109  36235‒36216 | 2 / 1  1 | 148 | Tierney et al, 1994; Bell et al, 2006. |
| Latent membrane protein 2 alpha  (outer primers) | LMP2A | CGGGATGACTCATCTCAACACATA  GGCGGTCACAACGGTACTAACT | 166412‒166435  163‒142 | 1  3 | - | Bell et al, 2006 |
| Latent membrane protein 2 alpha  (inner primers) | LMP2A | ATGACTCATCTCAACACATA  CATGTTAGGCAAATTGCAAA | 166416‒166435  381‒362 | 1  2 | 149 | Tierney et al, 1994 |

Prior to the nested PCR, the first-strand cDNAs were examined for the presence and quantity of the housekeeping gene *GUSB* (glucuronidase beta, RefSeqGene: NG_016197.1) cDNA (product size of 101 bp) by real-time PCR, using the following primers: 5’- GAAAATACGTGGTTGGAGAGCTCATT-3’ and 5’-CCGAGTGAAGATCCCCTTTTTA-3’. The nested PCR was performed in two rounds: the first round at 55° C of the annealing for 35 cycles and the second round at 55° C of the annealing for 25 cycles. Two microliters of the first-round PCR products were used as a template in the second round. The all samples have been assayed by nested PCR in at least two independent experiments. The all primer positions within the EBV strain B95-8 genome are shown in parentheses according to the NCBI GenBank Reference Sequence NC_007605.1.

**Table S2.** Demographic and clinical examination parameters in two groups of therapy-naïve CLL patients, in the EBV DNA-positive with the high avidity of anti-EBV-CA IgG and in the patients with the highest avidity of anti-EBV-CA IgG.

| Demographic and clinical parameters | Anti-EBV-CA IgG, RAI < 95.0  (range: 65.1–94.6)  EBV-positive  (n=9) | Anti-EBV-CA IgG, RAI > 95.0  (range: 96.2–131.3)  EBV-pos (n=9) and EBV-und (n=4) | *p*-value |
| --- | --- | --- | --- |
| Age (years)  Median (IQR: Q1–Q3): | Range: 41–88  69.0 (66–78) | Range: 52–85  68.0 (62–72) | > 0.05 |
| Gender: Male, number (%):  Female, number (%): | 3 (33.3)  6 (66.7) | 7 (53.8)  6 (46.2) | > 0.05 |
| Low-risk disease  (Rai stage 0), number (%): | 2 (22.2) | 7 (53.8) | > 0.05 |
| Intermediate-risk disease  (Rai stages I–II), number (%): | 5 (55.6) | 5 (38.5) | > 0.05 |
| High-risk disease  (Rai III–IV), number (%): | 2 (22.2) | 1 (7.7) | > 0.05 |
| TTFT (months)  Median (IQR: Q1–Q3): | Range: 0.0–21.5  0.3 (0.0–1.0) | Range: 0.0–21.5  0.5 (0.0–6.0) | > 0.05 |
| Leukocyte blood count (10^9/L)  Median (IQR: Q1–Q3): | Range: 10.2–106.6  28.1 (18.1–43.0) | Range: 11.6–71.9  19.7 (13.7–29.6) | > 0.05 |
| Lymphocyte blood count (10^9/L)  Median (IQR: Q1–Q3): | (n=8)  Range: 7.7–81.0  22.4 (13.3–58.0) | Range: 7.9–60.1  15.9 (11.4–24.2) | > 0.05 |
| Monocyte blood count (10^9/L)  Median (IQR: Q1–Q3): | (n=8)  Range: 0.2–6.9  2.2 (0.9–3.1) | (n=12)  Range: 0.3–2.4  0.7 (0.5–1.0) | > 0.05 |
| Neutrophil blood count (10^9/L)  Median (IQR: Q1–Q3): | (n=8)  Range: 2.2–12.0  3.8 (3.5–5.5) | Range: 1.1–7.3  3.2 (2.4–3.5) | > 0.05 |
| Platelet blood count (10^9/L)  Median (IQR: Q1–Q3): | Range: 116.0–269.0  217.0 (140.0–226.0) | Range: 120.0–252.0  170.0 (141.0–219.0) | > 0.05 |
| Red blood cell count (10^12/L)  Median (IQR: Q1–Q3): | Range: 3.6–5.4  4.0 (3.8–4.8) | Range: 1.1–5.0  4.1 (3.8–4.3) | > 0.05 |
| Hemoglobin (g/L)  Median (IQR: Q1–Q3): | Range: 103.0–167.0  123.0 (121.0–140.0) | Range: 45.0–154.0  125.0 (124.0–132.0) | > 0.05 |
| CD19+ cells in PBL (%)  Median (IQR: Q1–Q3): | Range: 69.0–94.0  87.0 (77.8–90.0) | Range: 66.0–97.1  82.7 (72.5–90.0) | > 0.05 |
| CD5+ in CD19+ PBL (%)  Median (IQR: Q1–Q3): | Range: 42.0–93.0  79.6 (65.0–83.0) | Range: 26.0–94.1  71.8 (54.5–83.0) | > 0.05 |
| CD23+ cells in PBL (%)  Median (IQR: Q1–Q3): | Range: 24.8–83.1  79.4 (72.2–81.8) | Range: 51.0–85.3  62.5 (56.0–74.0) | > 0.05 |

EBV-pos, the EBV-positive patient group, with the viral load >5 EBV DNA copies per 10^5 PBMCs that has been determined using the commercial quantitative real-time PCR kit; EBV-und, the EBV DNA-undetectable patient group, with the EBV load <5 EBV DNA copies per 10^5 PBMCs, (the threshold level stated in the kit); PBL, peripheral blood lymphocytes; PBMC, peripheral blood mononuclear cells. Anti-EBV-CA IgG avidity, the avidity level of the IgG antibodies against the EBV capsid antigen (EBV-CA); RAI, relative avidity index. TTFT, the time to the first treatment in months, with the following-up period of 60 months. The median values with interquartile ranges (IQR: 25% percentile and 75% percentile) and the absolute ranges (minimum–maximum) are presented. To compare differences between two groups of CLL patients, the Mann–Whitney test (including outliers) with the level of significance *p* < 0.05 was applied. Values of statistically significant differences between the groups are shown in Bold.

**Table S3**. Demographic and clinical examination parameters of therapy-naïve CLL patients with high EBV DNA load in PBMCs (n=3) and with the detected EBV transcripts, BZLF1 (n=1), LMP1 and EBNA2 (n=2).

| Parameters | EBV-pos / EBV-und | High EBV DNA load: (copies / 10^5 PBMCs) | | | LMP1 transcript-positive patients | |
| --- | --- | --- | --- | --- | --- | --- |
|  | Median |  |  |  | LMP2A– | LMP2A– |
|  |  |  | BZLF1+ |  | EBNA2+ | EBNA2+ |
| EBV copies / 10^5 PBMCs: | 16.0 / <5 | 264 | 258 | 523 | 6 | 6 |
| Patient No |  | 152 | 164 | 229 | 170 | 207 |
| Age in years | 68.0 / 70.0 | 69 | 72 | 77 | 64 | 41 |
| Gender: Male (%)  Female (%) | 42.9 / 63.6  57.1 / 36.4 | F | M | M | F | M |
| Rai clinical stage:  Low-risk (%):  Intermediate-risk (%):  High-risk (%): | 28.6 / 54.6  52.4 / 27.3  19.1 / 18.2 | Intermediate-risk | Low-risk | Intermediate-risk | Intermediate-risk | Intermediate-risk |
| TFTT, months | **0.0 / 1.0** | 1.0 | 3.5 | 0.0 | 0.0 | 0.0 |
| Leukocyte blood count (10^9/L) | 22.0 / 20.5 | 16.9 | 29.3 | 20.2 | 12.9 | 28.1 |
| Lymphocyte blood count (10^9/L) | 20.0 / 15.4 | 11.4 | 22.7 | 18.0 | 8.4 | 22.0 |
| Monocyte blood count (10^9/L) | 1.0 / 0.7 | 1.0 | 2.5 | NA | 0.9 | 2.0 |
| Neutrophil blood count (10^9/L) | 3.6 / 3.9 | 3.6 | 3.8 | 1.3 | 3.4 | 3.8 |
| Platelet blood count  (10^9/L) | 175.0 / 167.0 | 269.0 | 133.0 | 120.0 | 159.0 | 140.0 |
| Red blood cell count (10^12/L) | **4.1 / 4.4** | 4.0 | 3.8 | 3.4 | 4.2 | 4.8 |
| Hemoglobin (g/L) | **125.0 / 137.0** | 121.0 | 121.0 | 102.0 | 124.0 | 140.0 |
| CD19+ cells in PBL (%) | 82.8 / 83.2 | 69.0 | 90.0 | 82.7 | 85.0 | 77.8 |
| CD5+ cells in CD19+ PBL (%) | 72.8 / 58.3 | 62.0 | 88.3 | 83.0 | 54.5 | 65.0 |
| CD23+ cells in PBL (%) | 72.2 / 58.4 | 45.0 | 81.8 | 74.0 | 55.0 | 24.8 |

EBV-pos, the EBV-positive patient group, with the viral load >5 EBV DNA copies per 10^5 PBMCs that has been determined using the commercial quantitative real-time PCR kit; EBV-und, the EBV DNA-undetectable patient group, with the EBV load <5 EBV DNA copies per 10^5 PBMCs, (the threshold level stated in the kit). EBV-pos / EBV-und, the median values for both CLL patient groups, the EBV DNA-positive and the EBV DNA-undetectable, are presented and the values of statistically significant differences between the groups are shown in Bold. PBL, peripheral blood lymphocytes; PBMC, peripheral blood mononuclear cells. TTFT, the time to the first treatment in months with the following-up period of 60 months. In PBMCs of patient No 164 (with 258 EBV copies), the transcript of the EBV early lytic gene BZLF1 have been detected at the time of enrolment and the flow cytometry analysis. Patient No 229 (with 523 EBV copies), 18 months after the diagnosis with CLL, was diagnosed with the aggressive sebaceous carcinoma of the scalp with bilateral and abdominal metastases (T2N3M1 stage IVC); the patient underwent surgery and radiotherapy and died 24 months after the enrollment.

**5. References to the Supplementary Material**

1. Agathangelidis A, Sutton LA, Hadzidimitriou A, et al. Immunoglobulin Gene Sequence Analysis In Chronic Lymphocytic Leukemia: From Patient Material To Sequence Interpretation. J Vis Exp. 2018;(141):10.3791/57787. doi:10.3791/57787
2. Bell AI, Groves K, Kelly GL, et al. Analysis of Epstein-Barr virus latent gene expression in endemic Burkitt's lymphoma and nasopharyngeal carcinoma tumour cells by using quantitative real-time PCR assays. J Gen Virol. 2006;87(Pt 10):2885-2890. doi:10.1099/vir.0.81906-0
3. Gladkikh AA, Potashnikova DM, Tatarskiy V Jr, et al. Comparison of the mRNA expression profile of B-cell receptor components in normal CD5-high B-lymphocytes and chronic lymphocytic leukemia: a key role of ZAP70. Cancer Med. 2017;6(12):2984-2997. doi:10.1002/cam4.1257
4. Hallek M, Cheson BD, Catovsky D, et al. iwCLL guidelines for diagnosis, indications for treatment, response assessment, and supportive management of CLL. Blood. 2018;131(25):2745-2760. doi:10.1182/blood-2017-09-806398
5. Kholodnyuk I, Rivkina A, Hippe L, et al. Chemokine Receptors CCR1 and CCR2 on Peripheral Blood Mononuclear Cells of Newly Diagnosed Patients with the CD38-Positive Chronic Lymphocytic Leukemia. J Clin Med. 2020;9(7):2312. Published 2020 Jul 21. doi:10.3390/jcm9072312
6. Neves M, Marinho-Dias J, Ribeiro J, et al. Characterization of Epstein-Barr virus strains and LMP1-deletion variants in Portugal. J Med Virol. 2015;87(8):1382-1388. doi:10.1002/jmv.24190
7. Tierney RJ, Steven N, Young LS, Rickinson AB. Epstein-Barr virus latency in blood mononuclear cells: analysis of viral gene transcription during primary infection and in the carrier state. J Virol. 1994;68(11):7374-7385. doi:10.1128/JVI.68.11.7374-7385.1994
